# Assessment of residual tumour by FDG-PET: conventional imaging and clinical examination following primary chemotherapy of large and locally advanced breast cancer

**DOI:** 10.1038/sj.bjc.6605427

**Published:** 2009-11-17

**Authors:** J Dose-Schwarz, R Tiling, S Avril-Sassen, S Mahner, A Lebeau, C Weber, M Schwaiger, F Jänicke, M Untch, N Avril

**Affiliations:** 1 Department of Gynecology, Universitätsklinikum Hamburg-Eppendorf, Hamburg, Germany; 2 Department of Nuclear Medicine, Universitätsklinikum Hamburg-Eppendorf, Hamburg, Germany; 3 Department of Nuclear Medicine, Ludwig-Maximilians Universität, Munich, Germany; 4 Department of Nuclear Medicine, Technische Universität München, Munich, Germany; 5 Department of Pathology, Technische Universität München, Munich, Germany; 6 Department of Pathology, Universitätsklinikum Hamburg-Eppendorf, Hamburg, Germany; 7 Department of Gynecology, Ludwig-Maximilians Universität, Munich, Germany; 8 Department of Diagnostic and Interventional Radiology, Universitätsklinikum Hamburg-Eppendorf, Hamburg, Germany; 9 Department of Nuclear Medicine, Barts and the London School of Medicine, London, UK

**Keywords:** breast cancer, primary systemic therapy, FDG-PET, breast imaging, assessment of treatment response

## Abstract

**Background:**

The aim of this was to evaluate FDG-PET (2-(fluorine-18)-fluoro-2-deoxy-D-glucose positron emission tomography) for assessment of residual tumour after primary chemotherapy of large and locally advanced breast cancer in comparison with conventional imaging modalities.

**Methods:**

In a prospective multicentre trial, 99 patients underwent one or more breast imaging modalities before surgery in addition to clinical examination, namely, FDG-PET (*n*=89), mammography (*n*=47), ultrasound (*n*=46), and magnetic resonance imaging (MRI) (*n*=46). The presence of residual tumour by conventional imaging, dichotomised as positive or negative, and the level of FDG uptake (standardised uptake values, SUV) were compared with histopathology, which served as the reference standard. Patients with no residual tumour or only small microscopic foci of residual tumour were classified as having minimal residual disease and those with extensive microscopic and macroscopic residual tumour tissue were classified as having gross residual disease.

**Results:**

By applying a threshold SUV of 2.0, the sensitivity of FDG-PET for residual tumour was 32.9% (specificity, 87.5%) and increased to 57.5% (specificity, 62.5%) at a threshold SUV of 1.5. Conventional imaging modalities were more sensitive in identifying residual tumour, but had a low corresponding specificity; sensitivity and specificity were as follows: MRI 97.6 and 40.0%, mammography 92.5 and 57.1%, ultrasound 92.0 and 37.5%, respectively. Breast MRI provided the highest accuracy (91.3%), whereas FDG-PET had the lowest accuracy (42.7%).

**Conclusions:**

FDG-PET does not provide an accurate assessment of residual tumour after primary chemotherapy of breast cancer. Magnetic resonance imaging offers the highest sensitivity, but all imaging modalities have distinct limitations in the assessment of residual tumour tissue when compared with histopathology.

An increasing number of breast cancer patients are undergoing pre-operative (neoadjuvant) chemotherapy regimens. Primary systemic chemotherapy frequently reduces the tumour volume, which increases the frequency of successful breast-conserving surgery (van der Hage *et al*, 2001). Histopathology obtained from breast surgery after completion of chemotherapy serves as the reference standard for evaluation of residual tumour. Patients with no residual invasive tumour have longer disease-free and overall survival rates compared with patients with residual invasive tumour (Feldman *et al*, 1986; Machiavelli *et al*, 1998; Kuerer *et al*, 1999; Wolmark *et al*, 2001; Valero *et al*, 2002). However, only ∼10–25% of breast cancer patients achieve a histopathological complete response after primary systemic therapy (Bonadonna *et al*, 1998; Fisher *et al*, 2002; Bear *et al*, 2006). Other histopathological response classifications combine patients with no residual tumour and those with only small microscopic foci of residual tumour as minimal residual disease (MRD) compared with gross residual disease (GRD), which is defined as extensive residual microscopic or macroscopic tumour.

The accurate pre-operative assessment of residual tumour is important for guiding the surgical approach to ensure negative resection margins and to minimise morbidity. Different response patterns of primary breast tumours have to be taken into account, as some shrink concentrically to a solitary residual mass, whereas others leave scattered microscopic or macroscopic tumours within the tumour bed (Abraham *et al*, 1996; Partridge *et al*, 2002). Surgery focussing on the residual mass carries a higher risk of leaving microscopic tumour tissue behind, which may necessitate further surgical interventions or predispose to local recurrences. Conversely, tumours with a pathological complete response (pCR) may have unnecessary large resections of the tumour bed. Therefore, clinical examinations, as well as mammography, ultrasound, and magnetic resonance imaging (MRI), are often used to evaluate the presence and the extent of residual tumour. It has been suggested that positron emission tomography (PET) using the radiolabelled glucose analogue 2-(fluorine-18)-fluoro-2-deoxy-D-glucose (FDG) can be used to assess the extent and localisation of tumour deposits for a large variety of tumours, including breast cancer (Avril *et al*, 2000; Fletcher *et al*, 2008; Mahner *et al*, 2008). In lymphoma patients, FDG-PET has been recommended for routine post-treatment assessment, particularly for the differentiation between viable tumour and fibrosis and scarring in residual masses (Juweid *et al*, 2007).

The role of imaging modalities for post-chemotherapy assessment of primary breast cancers still needs to be defined. Breast ultrasound, mammography, and clinical examination tend to overestimate the residual tumour volume because of chemotherapy-induced necrosis and fibrosis (Yeh *et al*, 2005). Magnetic resonance imaging of the breast has been shown to be a sensitive method for visualisation of residual tumour, but the negative predictive value seems to be limited (Abraham *et al*, 1996; Rieber *et al*, 2002; Wasser *et al*, 2003; Denis *et al*, 2004; Warren *et al*, 2004; Belli *et al*, 2006; Segara *et al*, 2007). There is little information available on the potential role of FDG-PET in the assessment of residual breast cancer following primary chemotherapy.

The aim of our prospective multicentre study was to evaluate FDG-PET for monitoring primary chemotherapy in newly diagnosed large (⩾3 cm) or locally advanced breast cancer. Patients underwent FDG-PET at baseline, after the first and second cycle of chemotherapy and before surgery. We have recently reported our findings regarding the ability of early changes in tumour glucose metabolism to predict treatment response (Schwarz-Dose *et al*, 2009). In a previous publication, we analysed relative changes in tumour FDG uptake early in the course of neoadjuvant chemotherapy and compared the derived early metabolic tumour response with histopathological response after the completion of chemotherapy (Schwarz-Dose *et al*, 2009).

The analysis presented in this paper is distinctly different, as it addresses the role of FDG-PET for assessment of residual tumour after completion of pre-operative chemotherapy in comparison with conventional breast imaging modalities (mammography, ultrasound, MRI) and clinical examination. The presence of residual tumour, dichotomised as positive or negative, and the level of FDG uptake (standardised uptake values, SUV) were compared with histopathology, which served as the reference standard. In addition, the size of residual tumour measured by means of conventional breast imaging and assessed by clinical examination was compared with the size obtained from histopathology.

## Materials and methods

### Patients

Patients with newly diagnosed large (⩾3 cm) or locally advanced (UICC Stage III) non-inflammatory breast cancer, who participated in a prospective, randomised, multicentre trial comparing two regimens of pre-operative chemotherapy (epirubicin and paclitaxel either as standard dose (ET) or dose dense sequential regimen (EP) plus adjuvant cyclophosphamide, methotrexate, and fluorouracil (CMF)), were eligible for a prospective FDG-PET treatment monitoring study (Schwarz-Dose *et al*, 2009; Untch *et al*, 2009).

The Universitätsklinikum Hamburg-Eppendorf (UKE), the Ludwig Maximillians Universität München (LMU), and the Technische Universität München (TUM) participated in the FDG-PET study. In these three study centres, 173 patients were recruited into the ET/CMF trial, of whom 104 patients participated in the FDG-PET study. A total of 99 patients had one or more imaging modalities, either FDG-PET, mammography, breast ultrasound, or MRI for pre-operative assessment of residual tumour tissue. Detailed patient characteristics are given in Table 1.

Patients with known diabetes mellitus were not included, in addition to those falling under the exclusion criteria defined for the ET/CMF chemotherapy trial. The study protocol prospectively defined the technical parameters, including timing for FDG-PET imaging and criteria for anatomical and PET image analysis for the participating centres before initiation of the study.

Details of the ET/CMF trial and the option to participate in the evaluation of functional FDG-PET imaging were explained by a gynaecological oncologist and by a nuclear medicine physician. Written informed consent was obtained from all patients. Patients who refused participation in the evaluation of FDG-PET were still eligible for the ET/CMF trial. The study protocol was approved by the local ethical committees of the University Hospitals in Munich and Hamburg. The evaluation of FDG-PET for pre-operative assessment of residual tumour tissue was funded by the Deutsche Krebshilfe.

### Neoadjuvant (pre-operative) chemotherapy

The ET/CMF trial compared two regimens: epirubicin plus paclitaxel standard dose and dose dense sequential regimen. The standard dose regimen (ET) consisted of four cycles of epirubicin (90 mg m^−2^) and paclitaxel (175 mg m^−2^), given every 3 weeks. The ET dose dense sequential consisted of three cycles of chemotherapy with epirubicin (150 mg m^−2^) every 2 weeks, followed by three cycles of paclitaxel (250 mg m^−2^) every 2 weeks, in combination with G-CSF (filgrastim). After completion of chemotherapy, all patients underwent breast-conserving surgery or mastectomy.

### Assessment of histopathological residual tumour

Histopathological response was determined as previously described by Honkoop *et al* (1998). Surgical specimens were cut into 0.5 cm-thick slices and evaluated for the presence of macroscopic tumour. Representative samples were taken from all areas of macroscopically visible tumour and resection margins, as well as from areas with marked fibrosis or scarring. All sections were microscopically analysed for the presence of residual tumour. Immunohistochemical staining with antibodies against cytokeratins was performed on selected sections to identify or verify tumour residues. Complete response (pCR) required additional sampling from macroscopically suspicious and uninvolved areas of the surgical specimens. In addition, the ‘tumour bed’ was identified by signs of tumour regression, such as necrosis, presence of macrophages, or marked fibrosis. Specimens with no residual invasive tumour were classified as having complete histopathological response (pCR). Specimens with only few scattered foci of microscopic residual invasive tumour (⩽2 mm) were classified as having minimal residual disease (pMRD). For the purpose of this analysis, pCR and MRD were summarised in a response category MRD. Gross residual disease comprised tumours showing macroscopic residual tumour or extensive residual tumour infiltration on microscopic examination. The presence of residual ductal *in situ* carcinoma did not influence the histopathological response assessment.

### FDG-PET imaging

Patients fasted for at least 6 h before injection of 280–420 MBq (∼10 mCi) F-18 FDG. The mean blood glucose level was 101.7±13.6 mg per 100 ml. After an uptake period of 45 min, patients were positioned prone, with both arms at their sides on the scanner couch. A gap in the scanner support ensured no (deformation) compression of the breast. Whole-body PET scanners (ECAT951R/31 (TUM), ECATExact47 (UKE), and ECATExactHR+ (LMU); Siemens, Knoxville, TN, USA) were used. Emission scans of the breast (2D mode), acquired in one bed position, were obtained from 45 to 60 min after tracer injection, followed by a transmission scan with germanium-68 rod sources. Emission data were corrected for random events, dead time, and attenuation; image pixel counts were calibrated to activity–concentration (Bq ml^−1^) and decay was corrected using the time of tracer injection as reference.

Regions of interest (ROIs) were placed semi-automatically in attenuation-corrected images. The tumour was first identified on pre-treatment FDG-PET and subsequently an ROI was placed in the tumour bed on post-treatment FDG-PET. The slice with the highest radioactivity concentration within the tumour was identified and a circular ROI with a diameter of 1.0 cm was placed in this area and in the directly adjacent slices. Standardised uptake values were calculated using the average (SUVmean) and maximum (SUVmax) activity values within the ROIs, normalised to the injected activity and patient's body weight. Analysis of PET scans was performed without knowledge of the results of other clinical studies.

FDG-PET results were obtained for two SUV thresholds: SUV 2.0 and SUV 1.5. A positive PET result was defined as an SUV equal to and above the threshold level. A negative PET result was defined as an SUV below the threshold level. Histopathology served as reference standard, as described above.

To assess a potential influence of the timing of FDG-PET imaging after completion of chemotherapy, the mean and standard deviations were compared for SUVs obtained before and after 7, 14, 21, and 28 days after the last day of chemotherapy.

### Conventional breast imaging

After completion of primary chemotherapy and before surgery, the study protocol of the ET/CMF trial included at least one conventional imaging procedure, namely, mammography, breast ultrasound, and/or MRI. Investigations were performed in the same manner as routine clinical imaging procedures and were analysed by experienced radiologists. The results were dichotomised as positive or negative for the presence of residual tumour. In the case of a positive result, the lesions were measured in at least two dimensions. These data were documented in the case record form at the time of imaging and no retrospective analysis has been performed.

In brief, for mammographic assessment, cranio-caudal and lateral-oblique views of both breasts were acquired, with additional compression and lateral views as required. Identified lesions were measured in bi-directional maximum dimension. Ultrasound was performed of all four quadrants of the breast. For MRI, multisequence and multiplanar images of both breasts were obtained before and after administration of intravenous contrast using appropriate breast coils. The maximum length of enhancing lesions was measured in transverse, coronal, and sagittal planes.

### Physical examination of the breast

All patients were assessed by physical examination by the gynaecological oncologist before surgery. The tumour-involved breast was assessed by palpation. In concordance with anatomical imaging, results were dichotomised as positive or negative for the presence of residual tumour.

### Statistical analysis

Data collection was centralised in one study centre (UKE). The detection of residual tumour by physical examination, conventional imaging or the level of FDG uptake above the threshold SUV was defined as a positive test result. Histopathological evaluation of residual tumour served as the reference, and the rate of true and false positive test results, as well as the sensitivity, specificity, and positive and negative predictive values were calculated accordingly. The Mann–Whitney test was used to compare quantitative parameters between groups of patients. Spearman's rank correlation coefficient (*ρ*) was used to describe correlations between quantitative parameters. Quantitative parameters are expressed as mean±one standard deviation (s.d.). All statistical tests were performed two-sided at a 5% level of significance.

## Results

A total of 99 out of 104 patients who participated in the FDG-PET monitoring study had at least one imaging procedure after completion of chemotherapy before surgery, in addition to clinical examination, namely, FDG-PET (*n*=89), mammography (*n*=47), ultrasound (*n*=46), and MRI (*n*=46). A total of 17 patients had MRD and 82 patients showed GRD on histopathological assessment. Out of 17 patients with MRD, two had initially positive margins after surgery; one achieved tumour-free margins after a second resection, and the other patient underwent mastectomy due to extensive ductal *in situ* carcinoma (DCIS). Five patients with MRD underwent mastectomy: two patients due to extensive DCIS, one due to a large residual mass at the time of surgery, and two on the basis of patient preference.

### FDG-PET imaging

In all, 89 patients underwent pre-operative FDG-PET imaging of the breast. Standardised uptake values calculated for maximum and average activity values within a tumour ROI showed a close correlation (*ρ*=0.91). The SUVmax was used for subsequent analysis. Out of 89 patients, 16 (18.0%) had MRD and 73 patients (82.0%) had GRD in histopathology. The mean of the SUVmax in MRD was 1.4±0.7 compared with 1.8±0.9 in GRD. The difference was statistically significant (*P*=0.01).

We assessed two specific SUV thresholds for identifying residual tumour. By applying a threshold SUV of 2.0, 26 FDG-PET studies were positive (SUV ⩾2.0) and 63 were negative for residual tumour. However, 49 studies were false negative by using this threshold, resulting in a sensitivity of 32.9% of FDG-PET to detect residual tumour, with a corresponding specificity of 87.5%. When applying a threshold SUV of 1.5, the sensitivity to detect residual tumour increased to 57.5% (specificity 62.5%).

We also assessed whether the level of FDG uptake was influenced by the time interval between the last cycle of chemotherapy and FDG-PET imaging. Pre-operative FDG-PET imaging in all 89 patients was performed 16.4±6.4 days after the last cycle of neoadjuvant chemotherapy, before surgery. The mean SUV of all primary breast carcinomas after the last cycle of chemotherapy was 1.7±0.9. There was no statistically significant difference in the level of FDG uptake in patients who underwent FDG-PET imaging within 7, 14, 21, or 28 days after completion of chemotherapy (*P*>0.3).

### Mammography

A total of 47 patients underwent pre-operative mammography. Out of these 47 patients, 7 (14.9%) had MRD and 40 patients (85.1%) had GRD in histopathology. A total of 40 patients showed residual tumour on mammography and 7 were negative for residual tumour. The sensitivity of mammography to detect residual tumour was 92.5% and specificity was 57.1%. Three mammographies were false positive, of which two cases showed masses of 1 and 2.5 cm on mammography and no or MRD of less than 1 mm in histopathology. One patient had a residual mass of 8 cm on mammography and no residual invasive tumour but extensive *in situ* carcinoma in the histopathological specimen. Mammography was false negative in three cases, which histopathologically showed residual invasive tumours between 2.5 and 7.5 cm. The detailed results are summarised in Table 2.

### Breast ultrasound

A total of 58 patients underwent pre-operative breast ultrasound. Out of 58 patients, 8 (13.8%) had MRD and 50 patients (86.2%) had GRD in histopathology. A total of 51 breast ultrasound studies were positive, of which five were false positive. Ultrasound of the breast showed residual tumour masses between 0.4 and 3.3 cm in five patients who had no or MRD in histopathology. Only one of these five false-positive cases had residual *in situ* carcinoma. Breast ultrasound was true negative in three patients and false negative in four cases, which histopathologically showed residual ductal invasive carcinoma of 0.5 and 2.5 cm in two cases and residual lobular invasive carcinoma of 7.5 cm in one case. The sensitivity of the ultrasound to detect residual tumour was 92.0%, with a corresponding specificity of 37.5%. When comparing anatomical imaging modalities, the ultrasound had the highest rate of false-positive results (62.5%) and the lowest overall accuracy (89.7%) in detecting residual tumour after completion of chemotherapy. The results are summarised in Table 2.

### Breast MRI

A total of 46 patients underwent pre-operative MRI of the breast. Of these 46 patients, 5 (10.9%) had MRD and 41 patients (89.1%) had GRD in histopathology. In all, 43 breast MRI studies were positive for residual tumour, of which three were false positive. These three patients showed residual masses between 0.4 and 2 cm on breast MRI and had no or MRD in histopathology. However, two of these three patients had residual *in situ* carcinoma in histopathology. One breast MRI study was false negative and had residual ductal invasive carcinoma of 2.5 cm in histopathology. The breast MRI detected residual tumour with a sensitivity of 97.6% and a specificity of 40.0%. When comparing all imaging modalities, MRI had the highest overall accuracy of 91.3% for identifying residual tumour. The results are summarised in Table 2.

### Tumour size assessed by conventional imaging *vs* histopathology

The tumour size as assessed by mammography, breast MRI, and breast ultrasound showed a low correlation with histopathological tumour size (*ρ*-values 0.27, 0.43, and 0.50, respectively). Comparing the different imaging modalities with each other showed a reasonable correlation for mammography *vs* MRI (*ρ*=0.83; *n*=20), mammography *vs* ultrasound (*ρ*=0.78; *n*=41), and MRI *vs* ultrasound (*ρ*=0.82; *n*=24).

One patient with a residual tumour of 7.5 cm, which was neither detected by mammography nor by ultrasound, had a lobular invasive carcinoma. Another patient who had a residual tumour of 2.5 cm, which was not detected by mammography, ultrasound, or MRI, had a ductal invasive carcinoma. In an additional eight patients with lobular invasive carcinoma, residual tumour was detected by either mammography, ultrasound, or MRI.

### Physical examination of the breast

All 99 patients underwent a physical examination of the breast before surgery. The presence or absence of residual tumour was assessed by palpation. A total of 83 patients were positive for residual tumour, of which 8 cases were false positive. These eight patients were estimated to have residual tumours between 1 and 5 cm by physical examination and showed no or MRD in histopathology. Two of these eight patients had residual carcinoma *in situ*. Physical breast examination was false negative in seven patients, which histopathologically showed residual invasive tumours between 2.5 and 7.5 cm. The sensitivity and specificity of the physical examination to detect residual tumour were 91.5 and 52.9%, respectively. When comparing all imaging modalities, physical examination had the highest rate of false-negative results (8.5%) in identifying residual tumour. The results are summarised in Table 2.

## Discussion

Our prospective multicentre trial showed that breast MRI provided the highest sensitivity (97.6%) for identifying residual tumour after completion of primary chemotherapy compared with mammography, ultrasound, and clinical examination. However, the corresponding specificity for abnormal masses identified on MRI was low (40%). Previous reports have suggested that breast MRI is more accurate for assessment of residual tumour than conventional imaging (Denis *et al*, 2004; Warren *et al*, 2004; Yeh *et al*, 2005; Bhattacharyya *et al*, 2008). Nevertheless, these studies also revealed that MRI was prone to underestimate the extent of small residual tumour in up to 39% of patients (Denis *et al*, 2004; Warren *et al*, 2004; Yeh *et al*, 2005). This might partially be explained by tumour regression with residual scattered viable tumour cells within normal or necrotic tissue. In our study, MRI was less accurate in predicting complete pathological response or MRD with a negative predictive value of only 66.7%, which is an important limitation. This underlines that surgery cannot be obviated on the basis of results from imaging procedures. This is in line with a previous report of 45 patients, in which microscopic residual disease was found in 95% of patients who had a complete response in breast MRI (Belli *et al*, 2006). A recent review of evidence-based clinical applications for breast MRI suggested that contrast-enhanced dynamic MRI might be more suitable for differentiating between fibrosis and viable residual tumour (DeMartini and Lehman, 2008). In addition, a comparison with baseline MRI obtained before initiation of therapy might improve the accuracy of assessing residual tumour.

When comparing conventional imaging modalities, ultrasound had the highest rate of false-positive findings, with a specificity of only 37.5%. A distinct limitation of mammography and breast ultrasound is the differentiation between viable tumour and post-treatment changes such as scarring and fibrosis when a residual mass is present. Both extensive fibrosis and residual DCIS accounted for the high rate of false-positive results in conventional imaging, which ranged in our study between 42.9 and 62.5%.

We found a poor correlation between tumour sizes assessed by pre-operative imaging compared with histopathological tumour size. This is in contrast to previous reports, which found correlation coefficients ranging from 0.70 to 0.98 for MRI (Esserman *et al*, 1999; Partridge *et al*, 2002; Rosen *et al*, 2003; Wasser *et al*, 2003; Martincich *et al*, 2004). However, a recent retrospective review of neoadjuvant chemotherapy trials from the MD Anderson Cancer Center also found a poor agreement between tumour size measurements by mammography or ultrasound and histopathology (Chagpar *et al*, 2006). The authors concluded that there is no evidence that mammography or ultrasound performs better than physical examination for measuring residual disease after chemotherapy (Chagpar *et al*, 2006). In fact, the large prospective trials of neoadjuvant chemotherapy to date, NSABP-18 and NSABP-27, have not incorporated imaging for assessing residual tumour before surgery, but have relied on physical examination (Wolmark *et al*, 2001; Bear *et al*, 2006). In our study, physical examination of the breast before surgery was equally effective for the detection of residual tumour with a sensitivity of 91.5% and a corresponding specificity of 52.9%.

An important aspect of primary systemic chemotherapy in breast cancer is the low rate of complete pathological responses ranging from 10 to 25% (Bonadonna *et al*, 1998; van der Hage *et al*, 2001; Fisher *et al*, 2002). In our study, 17 out of 99 patients (17%) had MRD or a complete absence of tumour residues on histopathological examination. The majority of patients (83%) had GRD. This setting favours conventional imaging procedures, which might in part explain sensitivities to detect residual masses in the range of 90% for mammography, ultrasound, and MRI. However, the limited specificity between 37.5% for breast ultrasound and 57.1% for mammography reveals that there are no specific criteria established for the further characterisation of residual abnormalities as benign or malignant.

The metabolic activity of residual masses after systemic therapy, assessed by FDG-PET, was found to be a clinically applicable surrogate marker for treatment efficacy in a variety of settings. However, a small series of breast cancer patients who had achieved a good clinical response after primary chemotherapy revealed less-promising results (Burcombe *et al*, 2002). None of the patients presented with increased FDG uptake at the primary tumour site before surgery, but 9 out of 10 patients had residual invasive carcinoma on histopathology, ranging from 2 to 20 mm in size. We found a significantly lower FDG uptake after chemotherapy in MRD (SUV 1.4±0.7) compared with GRD (SUV 1.8±0.9).

Subsequently, two defined thresholds of FDG uptake, SUV >1.5 and SUV >2.0, were assessed for identification of residual tumour. FDG-PET provided the highest specificity among all imaging modalities: 62.5% using a threshold SUV >1.5 and 87.5% for a threshold SUV >2.0. By applying these criteria, the sensitivity was only 32.9 and 57.5%, respectively. The low sensitivity could be partially attributed to the limited spatial resolution of FDG-PET, which is in the range of 4–6 mm. However, other factors such as ‘metabolic stunning’ of residual viable tumour tissue after chemotherapy might have also contributed to the low detection rate. It is noteworthy that we found no influence on the level of FDG uptake depending on the time interval between the last cycle of chemotherapy and FDG-PET imaging. According to the study protocol, patients should undergo surgery within 4–6 weeks after the last cycle of chemotherapy and only six patients had surgery more than 28 days after chemotherapy. Response criteria developed for lymphoma patients by an International Harmonization Project (IHP) recommend a wait of 6–8 weeks after the last cycle of chemotherapy to assess treatment response (Juweid *et al*, 2007). However, this would be difficult in the setting of primary chemotherapy in breast cancer in which subsequent surgery is an integral part of the multimodality treatment plan. Conversely, the time interval between the last cycle of chemotherapy and surgery might also affect histopathology findings, which serve as the reference standard. One could hypothesise that prompt surgery might identify histopathological residual tumour tissue, which is already determined to undergo apoptosis and would not have been detected at later time points. FDG-PET is frequently used with great success for post-treatment assessment in the metastatic setting, including in breast cancer (Mahner *et al*, 2008; Avril *et al*, 2009). The metabolic information from FDG-PET generally provides a reliable marker of tumour viability and treatment response, and has been validated in several trials using clinical follow-up and survival as reference. Our results in the setting of primary chemotherapy using histopathology as reference are to some extent in contrast to these observations. The inability of FDG-PET to identify small tumour deposits may have contributed to the low accuracy of FDG-PET in our study.

In lymphoma, the level of FDG uptake after systemic therapy was found to carry prognostic information (Spaepen *et al*, 2003; Juweid *et al*, 2007). Whether metabolically inactive residual breast cancer patients (42.5% of patients using a SUV threshold >1.5) carry a better prognosis compared with patients with metabolically active residual tumours remains to be determined. A comparison of FDG-PET results with disease-free and overall survival is required once outcome data become available to evaluate whether FDG-PET may help to further stratify the group of patients with residual disease after chemotherapy.

The question arises regarding the role of imaging procedures after primary systemic therapy of primary breast cancer. Pre-operative imaging would ideally provide the following information: (i) accurate differentiation between responder and non-responder; (ii) localisation and extent of residual tumour; and (iii) prognostic information, for example, for further treatment stratification. We showed that residual tumour size assessed after completion of neoadjuvant chemotherapy by mammography, breast MRI, and breast ultrasound showed only a weak correlation with histopathological tumour size assessment. No current imaging modality can exclude the presence of microscopic tumour residues; therefore, surgery and a subsequent histopathological evaluation of the surgical specimen cannot be obviated. Thus, the main use of pre-operative imaging is to guide surgical treatment planning. Complete histopathological response (pCR) is defined as the absence of invasive tumour and may therefore include patients with residual DCIS. However, the presence of residual DCIS needs to be taken into account while defining the extent of surgery, but DCIS cannot be reliably identified and distinguished from regressive changes either by conventional imaging or by FDG-PET. Health-care cost is also an important consideration while applying imaging procedures, although no convincing evidence exists as yet of their benefits outside clinical trials. However, in a recent study, 84% out of 31 patients were identified by MRI as potentially suitable candidates for breast conservation after chemotherapy. Of them, breast conservation was achieved in 90.5% and the low rate (9.5%) of re-operation for positive resection margins indicates a potential role of breast MRI in surgical treatment planning in selected cases (Bhattacharyya *et al*, 2008).

Certain limitations of our study need to be taken into account. Not all patients underwent all imaging procedures, as the study protocol only included at least one conventional imaging procedure at completion of chemotherapy before surgery. The results were dichotomised as positive or negative for the presence of residual tumour and one could have developed a more sophisticated analysis. However, we believe that this approach reduced the potential influence of multiple observers, which is unavoidable in a prospective multicentre trial.

In conclusion, FDG-PET does not allow for an accurate assessment of residual tumour after primary chemotherapy of breast cancer. Magnetic resonance imaging offers the highest sensitivity, but all imaging modalities have distinct limitations in the assessment of residual tumour tissue when compared with histopathology.

## Figures and Tables

**Table 1:** Patient characteristics (*n*=99)

*Age (years)*	
Median	50
Range	30–66
	
*Menopausal status (n)*	
Premenopausal	49
Postmenopausal	50
	
*cT before chemotherapy (n)*	
T2	39
T3	42
T4	15
Tx	3
	
*cN before chemotherapy (n)*	
N0	22
N1	56
N2	11
Nx	10
	
*Estrogen receptor status (n)*	
Positive	61
Negative	32
Not available	6
	
*Histology (n)*	
Invasive ductal	87
Invasive lobular	11
Invasive medullary	1
	
*Grading (n)*	
G2	52
G3	45
Not available	2
	
*Chemotherapy (n)*	
ET regimen	55
EP regimen	44
	
*Residual disease after chemotherapy (n)*	
Minimal residual disease	17
Gross residual disease	82

Abbreviations: ET regimen=epirubicin and paclitaxel; cT=clinical tumour stage according to TNM classification; cN=clinical lymph node status according to TNM classification; EP=3 cycles epirubicin followed by 3 cycles paclitaxel at intervals of 2 weeks; ET=4 cycles of combined epirubicin plus paclitaxel at intervals of 3 weeks.

**Table 2:** Assessment of residual tumour after completion of primary systemic chemotherapy

	**Mammography (*n*=47)**	**Breast MRI (*n*=46)**	**Breast ultrasound (*n*=58)**	**Physical examination (*n*=99)**	**FDG-PET threshold SUV 2.0 (*n*=89)**	**FDG-PET threshold SUV 1.5 (*n*=89)**
Positive (*n*)	40	43	51	83	26	48
Negative (*n*)	7	3	7	16	63	41
True positive (*n*)	37	40	46	75	24	42
False positive (*n*)	3	3	5	8	2	6
True negative (*n*)	4	2	3	9	14	10
False negative (*n*)	3	1	4	7	49	31
Sensitivity (%)	92.5	97.6	92.0	91.5	32.9	57.5
Specificity (%)	57.1	40.0	37.5	52.9	87.5	62.5
PPV (%)	92.5	93.0	90.2	90.4	92.3	87.5
NPV (%)	57.1	66.7	42.9	56.3	22.2	24.4
Accuracy (%)	87.2	91.3	84.5	84.8	42.7	58.4

Abbreviations: FDG-PET=2-(fluorine-18)-fluoro-2-deoxy-D-glucose positron emission tomography; MRI=magnetic resonance imaging; NPV=negative predictive value; PPV=positive predictive value; SUV=standardised uptake values.

A positive imaging test result was defined as the detection of residual tumour. Histopathology served as reference standard (minimal residual disease=no residual tumour; gross residual disease=residual tumour).
